# Evaluation of the national tuberculosis surveillance and response systems, 2018 to 2019: National Tuberculosis, Leprosy and Buruli Ulcer Control Programme, Abuja, Nigeria

**DOI:** 10.11604/pamj.2020.35.54.21493

**Published:** 2020-02-24

**Authors:** Ayi Vandi Kwaghe, Chukwuma David Umeokonkwo, Mabel Kamweli Aworh

**Affiliations:** 1 Nigeria Field Epidemiology and Laboratory Training Programme, Abuja, Nigeria; 2 National Tuberculosis, Leprosy and Buruli Ulcer Control Programme, Abuja, Nigeria; 3 Department of Veterinary and Pest Control Services, Federal Ministry of Agriculture and Rural Development, Abuja, Nigeria; 4 Department of Community Medicine, Alex Ekwueme Federal University Teaching Hospital Abakaliki, Ebonyi State, Nigeria

**Keywords:** Surveillance system evaluation, tuberculosis, leprosy, buruli ulcer, Nigeria

## Abstract

**Introduction:**

Nigeria is among the countries with high Tuberculosis (TB) burden by global rating signifying the relevance of TB surveillance system evaluation in improving performance and capacity of the existing system. Hence, this evaluation was conducted in order to determine the gaps and proffer solution to enhance the TB surveillance system performance.

**Methods:**

questionnaires were administered to eight key informants using face-to-face interview method; data obtained was analyzed. Total number of TB cases and estimated number of cases for year 2018 was obtained. Percentage of positive cases using the GeneXpert test for 6 months (January to June 2019) was obtained. Available documents and publications on the National Tuberculosis, Leprosy and Buruli Ulcer Control Programme (NTBLCP) were also sought for information.

**Results:**

the NTBLCP has over 5,300 TB service points and 1,602 microscopy Centre’s distributed across the country. Acceptance for the standard TB case definition was 100%, forms used are easy to fill and diagnosis is laboratory-based requiring specialized trainings for laboratory personnel. The system had 25% sensitivity, high data quality with 100% timeliness. The TB surveillance system is representative of all ages. The system was first designed as TB and Leprosy Control Programme but later Buruli ulcer was incorporated into the Programme. First quarter supervisory visits are skipped due to late funding and delayed budget approval. Major share of the funding comes from donor partners.

**Conclusion:**

the system is useful, representative, acceptable, has good data quality, timely, and sensitive. The system is stable but needs to be funded more by the government. There is need for early funding and budget approval to avoid skipping of supervisory visits due to funding challenges. The system is not simple due the various test that need to be conducted before, during and after treatment to detect and verify that the patient is cured. We recommend continuous training of health workers, routine monitoring and evaluation, integration of TB care and prevention into other health services programmes like HIV/AIDS and active case search at all levels to increase the sensitivity of the system. Speed up the process of integration of NTBLCP surveillance system with IDSR for data harmonization in the country.

## Introduction

Tuberculosis is a global treat and Nigeria is among the high TB, TB/HIV and DR-TB countries globally. The country ranks 7^th^ among the 30 high TB burden countries globally and 2nd in Africa, accounting for 4% of the estimated incidence cases globally [1]. In 2015, the global estimate for new (incident) TB cases was estimated at 10.4 million. People living with HIV accounted for 1.2 million (11%) of the incident cases [2]. An estimated 460,000 cases of tuberculosis occur in Nigeria annually [1]. TB prevalence among HIV/AIDS patients rose up to 27% due to increased association of TB with HIV/AIDS [3]. The TB incidence and mortality rates for the country is 219/100,000 and 39/100,000 population respectively. The treatment coverage for the country in 2016 was 24% making it the lowest TB treatment coverage rate globally [1]. Nigeria ranks 8^th^ among the 30 high Multidrug Resistance (MDR) TB burden countries with a drug resistance TB prevalence of 4.3% among new cases and 25% among previously treated cases. A recent national survey by the National Tuberculosis, Leprosy and Bruli-ulcer Control Programme on catastrophic cost experienced by TB patients in Nigeria in 2017 reports that 51% of TB patients experienced catastrophic cost with a higher proportion (93%) among drug resistance TB patients by the human capital catastrophic cost estimation threshold of 20% [1] indicating the severity of TB in the country.

Public health surveillance systems are instituted for data collection, analysis, interpretation and dissemination of information to relevant authorities for action to improve public health [4, 5]. Currently, the human, animal and environmental health have instituted surveillance systems which are progressively being integrated into the One Health (OH) approach component [4]. Surveillance system evaluation enhances efficient utilization of data collection resources and optimizes the systems operation. It defines the usefulness of a specific system for a particular public health initiative based on the goals of the public health program and the data collection objectives [6]. The aim of surveillance system evaluation is to assess whether the system achieves the purpose and objectives of the programme [6]. The variation of public health surveillance systems in their methods, scope, purpose and objectives brings about the disparity of what is important in the system being evaluated [7]. The need for effective surveillance systems has long been recognized with increasing international pressure to improve the effectiveness of these systems [8]. The capacity of surveillance systems to accurately describe patterns of diseases is of public health importance. Therefore, regular and relevant evaluations of these systems are critical in order to improve their performance and efficiency [9]. This study aimed at assessing the national tuberculosis surveillance and response systems to determine if the system meets its goals and objectives and to recommend way of improving the system capacities.

## Methods

**Study setting:** Nigeria established the National Tuberculosis and Leprosy Control Program (NTBLCP) in 1989 with the mandate of reducing the prevalence of TB to a level it no longer constitutes public health problem in the country [10]. The NTBLCP is a division under the department of Public Health situated in the Federal Ministry of Health responsible for policy development, tertiary care, mobilization and development of human and material resource and provision of technical support to State programmes. The programme is in charge of coordination relating to the control of TB, Leprosy and Buruli Ulcer. Currently, the operating guideline for the programme is the Sixth Edition (2015) of the “National Tuberculosis, Leprosy and Buruli Ulcer Management and Control Guidelines [11]. At the national level, NTBLCP has National Coordinator, the Programme Manager and the Monitoring and Evaluation (M&E) unit for data management [11]. The NTBLCP comprises of 12 units responsible for conducting various functions. They include; TB/HIV, Public Private Mixed Directly Observed Treatment-Short course (PPM DOTS), M&E, Laboratory, Logistics, Programmatic Management of Drug Resistant Tuberculosis (PMDT), Community Tuberculosis Care (CTBC), Childhood TB, Leprosy/Buruli Ulcer, Advocacy Communication and Social Mobilization (ACSM), Finance and Administrative Units [12].

**Figure 1:**
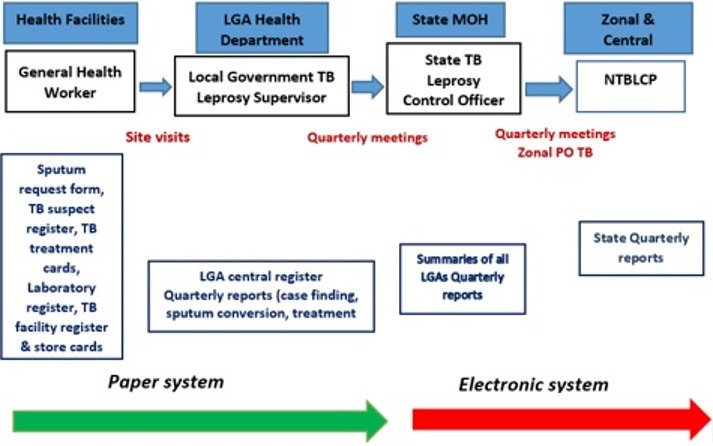
Current transmission path of TB data in Nigeria [12]

**Figure 2:**
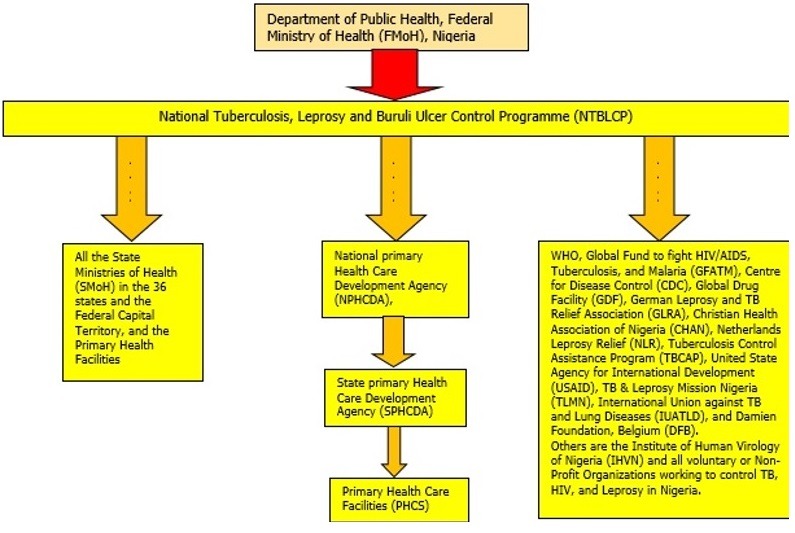
Stakeholders of NTBLCP, Nigeria [10, 20, 21, 22]

The program has three levels of its operation; federal, state and local governments. The State offices at each of the 36 State Ministries of Health across the country coordinate the activities of Local Government offices which in turn supervises the activities in the primary health facilities offering diagnostic and or treatment services. The health facility focal person known as the DOTS (Directly Observed Treatment Short course) Officer captures each presumptive patient in the TB presumptive register, after the preliminary test have been conducted, and the patient is confirmed TB positive, the patient details is now registered in the TB facility register. At the end of each quarter, the Local Government TB Supervisor goes round all the facilities under the LGA to aggregate the LGA summaries and harmonizes all the reports into the central register. This report is submitted to the State Officer during the state quarterly review meeting and subsequently to the national office. Electronic reporting of data starts at the state level (Figure 1). There exists a total of 774 LGA Supervisors in the country that report to the State Officers (37 State Officers which includes the FCT State Officer) and from the state to the national level [12]. The Zonal quarterly review meetings are held at the Zonal Headquarters with the State Officers based on the six geopolitical zones (Figure 1). The Zonal Headquarters are; Kaduna for North West, Nasarawa (North Central), Bauchi (North East), Enugu (South East), Ibadan (South West) and Port Harcourt (South South). Reports from the six zones are collected and collated at the national level for the production of quarterly report and presentations. The NTBLCP operates with the aid of stakeholders providing technical and financial support (details indicated in Figure 2 and Table 1).

**Table 1:** Major Technical and Financial Partners of the National Tuberculosis, Leprosy and Buruli-ulcer Control Programme

Stakeholder	Area of Work
USAID	Collaborative activities nationwide. USAID has been actively involved in 17 states in the northern part of the country, including Lagos and the FCT. USAID is also supporting the MDRTB survey and implementation of MDR-TB activities, including strengthening the zonal and national reference laboratories. USAID is also supporting the national TB logistic systems, community TB care, Public -Private Mix (PPM)-DOTS, and ACSM, and is strengthening the health system through renovation of facilities, human capacity building, and supply of microscopes and laboratory commodities. USAID has supported the development of a costing tool for provision of TB services, NTP strategic plan, and HRD plan 2010 -2015 and is strengthening supportive supervision through the use of person al digital assistants (PDAs).
WHO	Technical support in policy formulation, strategic planning, supervision, monitoring, and program evaluation at national and state levels. Resource mobilization and supply of anti-leprosy drugs.
Centers for Disease Control and Prevention (CDC)	Supporting IPs to provide TB/HIV collaborative activities in all the states of the federation and FCT. Support for the National MDR -TB survey and for diagnosis and management of MDR-TB, including culture, drug sensitivity test (DST) and polymerase chain reaction for line probe assay. QA system.
Damien Foundation Belgium (DFB)	Supporting TB and leprosy control in two states of S/W Nigeria (Oyo and Osun): provision of anti-TB drugs, lab reagents/consumables, training of health staff, and supervision. Supporting the MDR-TB Treatment Center at UCH-Ibadan (culture and DST, training, cost of patient management)
German Leprosy and TB Relief Association (GLRA)	Supporting TB and leprosy control in 14 states in S/E, S/W, an d S/S Nigeria (Abia, Akwa Ibom, Anambra, Bayelsa, Cross River, Delta, Ebonyi, Edo, Ekiti, Enugu, Imo, Ondo, Ogun, and Rivers): Provision of anti -TB drugs, microscopes, project vehicles, and general logistics (to 2006); lab reagents/consumables, training of heal th staff, and supervision. TB/HIV project in Lagos: coordinates ILEP support to leprosy control in Lagos state; piloted PPM in Anambra and Abia; operational research in TB, TB/HIV and leprosy; pilot project in Buruli Ulcer control in Cross River State.
Netherlands Leprosy Relief (NLR)	Supported TB control in four states in N/C and N/E Nigeria (Bauchi, Gombe, Kaduna, and Plateau): provision of anti -TB drugs, lab reagents/consumables, training of health staff and supervision (to 2006). Supporting 13 st ates in leprosy control (Adamawa, Bauchi, Benue, Borno, Gombe, Jigawa, Kaduna, Kano, Katsina, Nasarawa, Plateau, Taraba, and Yobe): provision of logistics (vehicles and motorcycles) for TB control in combination with leprosy.
The Leprosy Mission Nigeria	Supporting leprosy control in seven states of N/C and N/W Nigeria (FCT, Kebbi, Kogi, Kwara, Niger, Sokoto, and Zamfara). Partly supporting TB in those states with respect to training of state and LGA TBL supervisors and technical support in field supervision.
Private Health Care Providers	Provide service for profit (mainly clinical care). Current piloting to determine public–private sector mix in TB control.
Abt Associates Inc.	*Partnership for Transforming Health Systems 2 (PATHS2):* Designed to facilitate efficient use of Nigeria’s resources to attain the MDGs through improving the planning, financing, and delivery of sustainable and replicable pro-poor health services for common health problems in specific locations (Enugu, Jigawa, Kaduna, Kano, and two others to be determined). *Health Systems 20/20*: In collaboration with the Ministry of Health, piloting supportive supervision using PDAs National TB Services Improvement via Human Capital Development: Costed Plan Continued Improvement and Application of the Nigeria TB Direct Costing Tool Improved diagnostics for TB, in collaboration with Tulane University: will provide technical assistance to Zaria TB Institute to improve TB diagnostics (e.g., for under-diagnosed pediatric TB), with the new Microscopic Observation-Drug Susceptibility (MODS) technique for culturing Mycobacterium tuberculosis. Strengthening TB laboratory capacity, in collaboration with the South African Medical Research Council (SMRC): will support Zaria to enhance laboratories’ diagnostic and management systems by updating manuals/guides; creating a new overall framework for managing TB laboratory networks; helping to train laboratory personnel in new guidelines and frameworks; and introducing modern methods for TB detection and treatment, such as LED microscopy. Establishing systems for systematizing and harmonizing laboratory quality assurance
JSI Deliver (USAID | DELIVER PROJECT)	Providing technical assistance and capacity building in logistics management of TBLCP commodities. Main focus is on central -level capacity building, with support to Logistics Unit and NTBLTC in system development and implementation of capacity building for health care workers HCWs. Provision of technical support for implementation of Procurement and Supply management functions with the NTBLCP, including national -level quantification, supply planning, and inventory management.

**Source:** FMoH [10]

**Case definition:** a suspected pulmonary TB case is defined as any person coughing for 2 weeks or more, with or without symptoms of weight loss, tiredness, fever, night sweats, chest pain, shortness of breath, loss of appetite and coughing up blood while a suspected extra-pulmonary TB case is a person with symptoms depending on the affected organ, vertebral spine (back pain, swelling on spine); bone (long standing pain and swelling of the bone); Joints (painful joint swelling, usually affecting one joint); kidney and urinary tract (painful urination, blood in urine, frequent urination, lower back pain/loin pain); upper respiratory tract (hoarseness of voice, pain on swallowing); pleural membrane of lungs (chest pain, difficulty in breathing, fever); meninges of the brain (headache, persistent fever, neck stiffness, vomiting, irritability, convulsions, loss of consciousness); lymph node (painless swelling of the node, may drain pus); skin (longstanding ulcer despite antibiotic treatment, draining pus) and some general symptoms (weight loss, persistent fever, night sweats). In children, a suspect case is defined as any or combination of the following symptoms: low grade fever not responding to malaria treatment, night sweats, loss of weight, loss of appetite, failure to thrive, lymph node swellings, joint or bone swellings, angle deformity of the spine, listlessness and neck stiffness, headache, vomiting (TB meningitis) [13]. A confirmed TB case must be laboratory diagnosed. Case detection is done through screening of TB symptoms, along with sputum microscopy for AFB. Two positive smears out of three define positivity for TB. For sputum smear-negative cases, diagnosis is made after three negatives, along with X-ray and confirmation by medical officer. Extra-pulmonary TB cases in children are diagnosed by medical officers using clinical examination, X-ray, and/or any other investigation deemed necessary to establish a diagnosis [10].

**Study design and data collation:** we conducted a descriptive evaluation of the National TB surveillance system using Centers for Disease Control and Prevention (CDC) updated guidelines for evaluating public health surveillance systems [14] and the CDC Overview of Evaluating Surveillance Systems [6]. Questionnaire used for this study was adapted from the WHO generic questionnaire for central level (national), protocol for the assessment of national communicable disease surveillance and response [15] with minor modifications to suit the surveillance system being evaluated. The questionnaire had 9 sections, namely; socio-demographic characteristics; general questions; case definition, registration and sensitivity; representativeness; data reporting; data analysis; feedback; training; and surveillance. Questionnaires were administered to key informants from the PMDT, ACSM, TB/HIV, PPM DOTS, Childhood TB, Leprosy/Buruli Ulcer and M&E units of the NTBLCP, Abuja. A total number of 8 questionnaires were interviewed-administered. Selection was based on the roles that each key informant performs in the unit; directly linked with supervision, data collation and feedback.

**Data source and study duration:** we obtained national data for the 36 states and the Federal Capital Territory (FCT) from the M&E unit of the NTBLCP; total cases of drug susceptible TB in the country for the year 2018 and the estimated projected number of cases for the same year; data from January to June 2019 for the GeneXpert test (total number of cases that were tested using the GeneXpert test and the total number of positive cases). Data obtained was collated. This study was conducted at the national level Abuja, from 16^th^ September to 25^th^ October, 2019.

**Data analysis:** data obtained from questionnaires and key informant interviews were cleaned and analyzed. We presented the descriptive statistic and also determined the sensitivity and predictive value positive as well as the other attributes of the surveillance system.

**Ethical issues:** this study was conducted based on approval by the national coordinator of the NTBLCP after a letter for the release of data was sent. Secondary data was used for this study and no ethical clearance was required. Verbal consent of the key informants was obtained before the face-to-face interview was conducted.

## Results

**Usefulness of the system:** at the end of 2014, there were over 5,300 TB service points and 1,602 microscopy Centre’s distributed across the entire country [11]. The system detects TB in health facilities from the 774 local government areas of the federation nationwide. Gene Expert machines are used in the detection of drug resistant tuberculosis nationwide. For new cases where there are no GeneXpert machines, AFB test are used to diagnose TB. It is mandatory for re-occurring cases to use GeneXpert test to detect DR-TB cases. Data obtained from the surveillance are used to measure performance of the system, determine the health indicators for needs assessments and accountability of the system. Aside TB detection, other diseases detected by the system are leprosy and buruli ulcer. Data is also shared with WHO (Table 2).

**Table 2:** Demographic characteristics, case definition, case registration, sensitivity and representativeness, N = 8

Categories	Variables	Items	Percentage
**Demographic characteristics**	Sex	male	50.0
		female	50.0
	Marrital Status	Married	75.0
		Single	25.0
	Staff Cadre	Doctors	37.5
		medical scientist	12.5
		Others	50.0
	Working Experince	<5 years	0.0
		5-9 yesra	37.5
		10-14 years	0.0
		>15 years	62.5
**General**	Mandatory TB Surveillance?	Yes	100.0
		No	0.0
	TB manual?	Yes	100.0
		No	0.0
**Case Definition, Registration and Sensitivity**	TB Case definition?	Yes	100.0
		No	0.0
	Use case definition for TB detection?	Yes	100.0
		No	0.0
	System detect all TB cases?	Yes	12.5
		No	87.5
	Frequent cases of misdiagnosis?	Yes	25.0
		No	75.0
	Suggestions for improvement	Training and re-training of health workers, monitoring and evaluation.	75.0
		Integration of TB care and prevention into other health services and programmes like HIV/AIDS	12.5
		Active case search at all levels	12.5
**Representativeness**	System capture children < or > 15 years of age?	Yes	100.0
	Capture children nationwide?	Yes	100.0

### System attributes

**Simplicity:** forms used are easy to fill and diagnosis is strictly laboratory-based. Some of the test carried out are expensive and require ed case definition includes a TB positive result from one of the test procedures. The tests conducted are AFB microscopy (Ziehl-Neelsen technique), culture, GeneXpert^®^ MTB/RIF assay, liquid culture using the Mycobacterium Growth Indicator Tube (MGIT), which allows rapid growth and detection of *M. tuberculosis* and polymerase chain reaction (PCR) using strip technology in line probe assay (LPA) for drug susceptibility testing (DST). Others include, quantiferon test used for latent diagnosis of TB, Lateral Flow Urine Lipoarabinomannan Assay (LF-LAM) for the diagnosis and screening of active TB in people living with HIV, Loop-Mediated Isothermal Amplification (TB-LAMP) for the diagnosis of pulmonary tuberculosis and in follow-up cases. Data is evaluated in terms of person, place and time. Demographic data obtained from WHO and the National Population Commission which is used in the calculation of rates and surveillance data from the NTBLCP (Table 3). The system of reporting at the central level is computerized, has a central data based server, data storage and analysis. The system is linked with the electronic TB (E-TB) manager for alerting of new TB cases and the GXalert messages for detected DR-TB cases from GeneXpert^®^ MTB/RIF assay machine (Table 3).

**Table 3:** Data reporting, data analysis, feedback, training and surveillance, N= 8

Categories	Variables	Items	Percentage
**Data Reporting**	Central distribution of forms?	Yes	100.0
	Lack surveillance forms in past 6months?	Yes	37.5
		No	62.5
	FMoH share data with WHO?	Yes	100.0
	Reporting method	Electronic	100.0
**Data Analysis**	Data by person?	Yes	100.0
	Data by age & sex?	Yes	100.0
	Data by Place?	Yes	100.0
	Data by district (tables& maps)?	Yes	100.0
	Data by time?	Yes	100.0
	Trend analysis?	Yes	100.0
	Action threshold?	Not Applicable	100.0
	TB in eradication process?	Yes	25.0
		No	75.0
	TB epidemic prone?	Yes	12.5
		No	87.5
	Demographic data?	Yes	100.0
	Rates from demographic data?	Yes	100.0
	Source of demographic data?	WHO	50.0
		NPC	50.0
**Feedback**	Editorial board at FMoH?	Yes	62.5
		Unknown	37.5
	Editor?	Yes	62.5
		Unknown	37.5
	Budget for publication?	No	12.5
		Unknown	87.5
	Feedback mechanism?	Annual Bulletin	100.0
		Quarterly Zonal Presentations	100.0
	Supervision for the past 6 months?	Yes	100.0
		How many visits done?	16(66.7)
		Obtained visits from central level?	24(100)
**Training**	Personnel trained in surveillance?	Yes	75.0
	Trained in disease surveillance?	Yes	62.5
		No	37.5
	Post basic training in disease surveilance?	Yes	37.5
		No	62.5
	National Epid. /PH Society?	Yes	62.5
		Unknown	37.5
**Surveillance**	Computerized surveillance network?	Yes	100.0
	Links with other levels?	Yes	100.0

**Representativeness:** the TB surveillance system is representative of all ages. Children below and above the age of 15 years are covered nationwide (Table 2). There is regimen for the treatment of children that are diagnosed with TB. Nationwide all age group and every district is under surveillance. Therefore, data obtained from the surveillance is representative of the entire national population. Data obtained is described in terms of person (sex and age), place (distribution using tables, charts and maps) and time (trend analysis and period of occurrence), (Table 3).

**Acceptability:** there exist TB manual which includes other diseases; leprosy and buruli ulcer. The standard TB case definition is well accepted (100%) by the staff (Table 1). There was full participation of all stakeholders in the programme and a good public-private partnership with the programme.

**Flexibility:** the system was first designed for TB and Leprosy Programme but later on Buruli Ulcer was incorporated into the Programme. Presently, the system surveillance constitutes TB, Leprosy and BU supervisors (TBLS) in all the LGAs and State TB programme Managers in all the States of the country. Currently, discussions between the NTBLCP and the Integrated Disease Surveillance and Response (IDSR) for integrating TB data from NTBLCP into the IDSR for harmonization of the national TB data is ongoing.

**Data quality:** case definition is consistent with WHO guidelines. The TB surveillance system was designed to capture a minimum set of variables for reported TB cases which was met by evaluation (core variables are captured; age, sex, case type, distribution) (Table 3). All scheduled periodic data submissions received and processed at the national level. Data are currently captured in an appropriate aggregated database for further analysis. At the central level, data is analyzed and interpreted collectivity to evaluate the national state of TB and TB trend in various states. The central level provides feedback to the states on quarterly basis at the zonal review meetings in the zonal office of each geopolitical zone in the country (Table 3). Power point presentations are delivered and information is shared with the state epidemiologist who in turn disseminate the information to LGA supervisors and subsequently to the health facilities concerned (Table 3).

**Timeliness:** timeliness was not assessed on weekly or monthly basis because at the central level, reports are compiled on quarterly basis for quarterly zonal meetings presentations. Timeliness was scored at 100% because all the reports are always made available at the stipulated time before the quarterly review meetings.

**Sensitivity and predictive value positive (PVP):** the projected number of cases for NTBLCP for the year 2018 was 419,000 out of which 104, 904 cases (25%) were detected by the system. A total of 285, 717 patients were tested using the GeneXpert test to detect DR-TB from January to June 2019, out of this number 36, 223 (12.7%) were positive. Hence, the sensitivity of the system was 25% while the PVP for DR-TB using the GeneXpert test was 12.7%. There were no frequent cases of misdiagnosis (Table 2).

Sensitivity = (Total number of positive cases in year 2018 (104,904))/(The projected number of cases by NTBLCP for the year 2018

(419,000))× 100 = 25.0%

PVP =(Total number of confirmed cases using GeneXpert test (36,223))/(Total number of cases submitted to the laboratory for GeneXpert test

(285,717))×100 = 12.7%

**Stability:** The NTBLCP has dedicated staff handling various thematic areas and an operational structure but limited funding from the government (budget from FMoH) that makes the programme mainly donor driven. The system has existed for 3 decades.

## Discussion

The TB surveillance system was found useful in the detection of drug susceptible and DR-TB cases as well as in evaluating the TB health indicators. This was based on the CDC assessment of usefulness of a surveillance system by its ability to determine adverse health event that contributes to performance measures such as health indicators that are used for need assessment and system accountability [16]. Data obtained has signified that the case definition is simple and easy to use but a confirmed case has to do with laboratory test which may be fairly easy or complex to conduct. This require specialized and trained personnel making the system a bit complex to operate. Test also need to conducted at the end of treatment to verify if the patient is cured or not. The simplicity of a public health surveillance system refers to both its structure and ease of operation. Surveillance systems should be as simple as possible while still meeting their objectives [14]. In 2018, 25% of the projected number of cases was detected by the surveillance system, indicating the need for improved active case search at all levels for early case detection to meet the objective and goal of the surveillance system in reducing the prevalence of TB to a level that no longer constitute public health problems in the country which will significantly diminish the socioeconomic impact and rate of transmission. The system is not yet integrated with other systems. Discussion on data integration and harmonization with IDSR is ongoing.

Integration and harmonization of data from NTBLCP and IDSR through collaboration will increase the validity of TB data obtained and distributed across the country and at international level. Differences in data obtained indicates a missing link between the two organizations. Hence, the dare need to harmonize data obtained. Data obtained has indicated that the TB surveillance system is representative of the TB cases in the country based on the CDC guideline that was used to evaluate the credibility of the system which states that a public health surveillance system is representative when it accurately describes the occurrence of a health-related event over time and its distribution in the population by place and person [16]. In 2016, at least one million children were estimated to have acquired tuberculosis annually, representing about 10% of all tuberculosis (TB) cases globally, while 250,000 children died of TB (including those co-infected with HIV) [17]. During the same period, there were an estimated 56,000 cases of TB in children in Nigeria [18] indicating that children are well captured in the TB surveillance system in the country. When cases notified to a surveillance system are not representative of the events in the general population, there is poor reflection of national and global situation of the disease [14].

Over time, the representativeness of surveillance data may change due to changes in legislation, surveillance infrastructure, clinical practice and reimbursement policies leading to wrong conclusions. For example, when trends in surveillance data are compared between countries. Hence the need for stability in the representativeness on the national surveillance level needs to be monitored, and the quantitative effect of changes on representativeness assessed [14]. There are 32 supervisory visits to be made in a year at the central level, 8 visits in each quarter but the visit in the first quarter is usually skipped due to late funding of the budget. Thus, the need for early funding to avoid the skipping of the first quarter supervisory visits. The system demonstrated stability based on reliability; signifying the systems´ ability to collect, manage, and provide data properly without failure and availability; ability to be operational when needed. However, the funding of the programme is mainly by the donor partners. This indicates that the Nigerian government needs be the major funder of the programme for sustained stability in the future because funding plays a major role in the performance and sustenance of a system. Peradventure there is withdrawal from any of the major donors in the future, this may lead to a major setback in the programme. Studies have indicated prospects for TB control in Nigeria with the existence of a 30 year old TB control programme and the willingness of development partners to assist [19].

**Limitation of the study:** evaluation of validity and available resources in terms of funding was not done.

## Conclusion

The system was found to be useful, representative, acceptable, good data quality, timely, and sensitive. The system is also stable but needs to be funded more by the government. The stability of the programme largely depends on the funding from donor partners which needs to be corrected. There is need for early funding to avoid the skipping of the first quarter supervisory visits. The system is not simple due the various test that need to be conducted before, during and after treatment to verify that the patient is cured. Recommendations include training and re-training of health workers, constant monitoring and evaluation of the TB surveillance system, integration of TB care and prevention into other health services and programmes like HIV/AIDS and active case search at all levels. Speed up the process of integration of NTBLCP surveillance system with IDSR for data harmonization and better output in case detection. Findings will be communicated to partners and stakeholders in order to improve on the existing TB surveillance system and response.

### What is known about this topic

- Surveillance system evaluation is conducted to improve public health;
- Evaluation is done based on the type of system instituted;
- The instituted system for surveillance should be able to meet its set goals and objectives.

### What this study adds

- The TB surveillance system was found to be useful, representative, acceptable, has good data quality, timely and sensitive. Nevertheless, 25% sensitivity of the surveillance system indicates the need for improved active case search at all levels for early case detection to meet the objective and goal of the surveillance system;
- There is need for government to increase funding of the TB surveillance system because majority of the funds is from donor partners;
- There is need for early funding and budget approval to avoid the skipping of the first quarter supervisory visits.

## Competing interests

The authors declare no competing interests.
